# Molecular mechanism for USP7-mediated DNMT1 stabilization by acetylation

**DOI:** 10.1038/ncomms8023

**Published:** 2015-05-11

**Authors:** Jingdong Cheng, Huirong Yang, Jian Fang, Lixiang Ma, Rui Gong, Ping Wang, Ze Li, Yanhui Xu

**Affiliations:** 1 Fudan University Shanghai Cancer Center, Institutes of Biomedical Sciences, School of Basic Medical Sciences, Shanghai Medical College of Fudan University, 131 Dong An Road, Mingdao Building, Room 715, Shanghai 200032, China; 2 Key Laboratory of Molecular Medicine, Ministry of Education, Department of Systems Biology for Medicine, School of Basic Medical Sciences, Shanghai Medical College of Fudan University, Shanghai 200032, China; 3 State Key Laboratory of Genetic Engineering, Collaborative Innovation Center of Genetics and Development, School of Life Sciences, Fudan University, Shanghai 200433, China; 4 Department of Anatomy and Histology & Embryology, Shanghai Medical College of Fudan University, Shanghai 200032, China

## Abstract

DNMT1 is an important epigenetic regulator that plays a key role in the maintenance of DNA methylation. Here we determined the crystal structure of DNMT1 in complex with USP7 at 2.9 Å resolution. The interaction between the two proteins is primarily mediated by an acidic pocket in USP7 and Lysine residues within DNMT1's KG linker. This intermolecular interaction is required for USP7-mediated stabilization of DNMT1. Acetylation of the KG linker Lysine residues impair DNMT1–USP7 interaction and promote the degradation of DNMT1. Treatment with HDAC inhibitors results in an increase in acetylated DNMT1 and decreased total DNMT1 protein. This negative correlation is observed in differentiated neuronal cells and pancreatic cancer cells. Our studies reveal that USP7-mediated stabilization of DNMT1 is regulated by acetylation and provide a structural basis for the design of inhibitors, targeting the DNMT1–USP7 interaction surface for therapeutic applications.

## Abstract (web summary)

DNMT1 is a methyl-transferase involved in maintaining tissue-specific patterns of DNA methylation. Here the authors solve the structure of a DNMT1-USP7 complex and demonstrate the mechanism by which DNMT1 stability is regulated through acetylation by preventing association with the deubiquitinase USP7.

## Graphical abstract

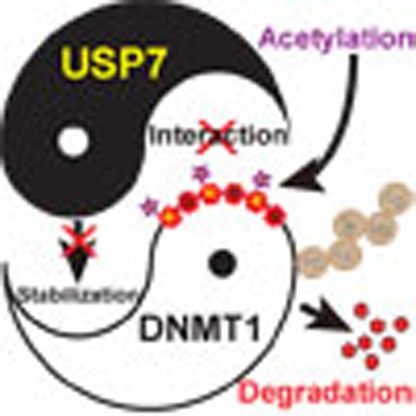

DNA methylation is a major epigenetic modification that plays an important role in several biological processes, including the regulation of transcription, chromatin structure, genomic imprinting and silencing of repetitive DNA elements^1^,^2^. In vertebrate, DNA methylation mainly occurs at the carbon 5 position of cytosine in a context of cytosine–guanine dinucleotide^3^. Aberrant DNA methylation is frequently observed in many human cancers and is known to contribute to tumorigenesis^4^.

Mammalian DNA methylation patterns are established by the *de novo* DNA methyltransferases DNMT3a and DNMT3b during embryonic development and are faithfully propagated to daughter cells by maintenance DNA methyltransferase DNMT1 during replication^5^. Ubiquitin-like (UBL), containing PHD and RING finger domains, 1 (UHRF1, also known as NP95 in mouse) binds to hemi-methylated DNA and histone H3K9 tri-methylation and recruits DNMT1 for chromatin localization^6^,^7^,^8^. It has been shown that DNMT1 is highly expressed in several cancer types and that overexpression of DNMT1 plays an important role in tumorigenesis^9^.

The expression of DNMT1 is regulated by a number of signalling pathways— including PI3/PKB, Rb/E2F and p53/SP1—at the transcriptional level, and the stability of DNMT1 is further regulated by post-translational modifications such as methylation, phosphorylation, acetylation and ubiquitination^10^,^11^,^12^. Previous studies also indicated that SET7 methylates human DNMT1 at residue K142 mainly during late S-phase and that methylation promotes proteasomal degradation of DNMT1 in a cell cycle-dependent manner^13^. Set7/9 methylates residue K1096 of mouse Dnmt1 and leads to decrease of stability, whereas the histone demethylase LSD1-mediated demethylation of K1096 is required for maintenance of DNA methylation and gastrulation during mouse embryogenesis^14^. HSP90 interacts with and stabilizes DNMT1, whereas acetylation of HSP90 disrupts the interaction and prompts degradation of DNMT1 (ref. 15). Recent studies show that the Ubiquitin-Specific Protease 7 (USP7, also known as HAUSP, the herpes virus-associated USP), binds to and regulates DNMT1 stability through acetylation and ubiquitination^16^,^17^,^18^. However, the molecular mechanism for USP7-mediated stabilization of DNMT1 remains largely unknown.

USP7 deubiquitinates several tumour suppressors (p53, PTEN, FOXO and claspin) and E3 ligases (MDM2, Mule and viral proteins ICP0) and therefore regulates important signalling pathways that are involved in tumorigenesis^19^,^20^. Interestingly, mounting evidence indicates that USP7 also deubiquitinates chromatin-associated proteins, including histone H2B, UHRF1 and Tip60 (refs 16, 17, 18, 21, 22, 23, 24). Thus, USP7 is implicated in tumorigenesis, DNA repair, immune response, viral invasion and epigenetic regulation^19^. Consistent with the above functions, USP7 is upregulated in many cancer cells, such as prostate, colon, bladder, liver and lung cancers^25^,^26^, and is believed to be a potential drug target^19^.

To explore the molecular mechanism for USP7-mediated stabilization of DNMT1, we determined the crystal structure of human DNMT1 in complex with USP7. Structural and biochemical analyses reveal that the interaction is mainly mediated by the KG linker of DNMT1 and a previously uncharacterized acidic pocket that acts as a substrate-binding site near the C-terminus of USP7. Mutations of these acidic residues disrupt the interaction between DNMT1 and USP7, leading to increased turnover of DNMT1. Acetylation of Lysine residues of the KG linker impairs the DNMT1–USP7 interaction and promotes proteasomal degradation of DNMT1. The anti-correlation of acetylated DNMT1 versus total DNMT1 was observed under physiological and pathological conditions. Overall, our studies provide new insights into the acetylation-regulated and USP7-mediated stabilization of DNMT1.

## Results

### Interaction between DNMT1 and USP7

**Figure 1:**
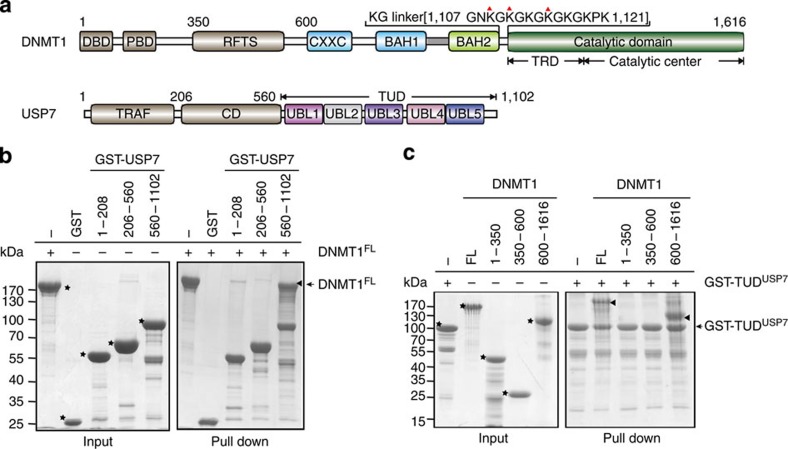
Interaction between DNMT1 and USP7. (**a**) Colour-coded domain architecture of human DNMT1 and USP7. The colour scheme is used in all structural figures. DBD: DMAP1 binding domain; PDB: PCNA binding domain; RFTS: replication foci-targeting sequence; CXXC: CXXC type zinc finger; BAH: bromo-adjacent homology domain; TRD: target recognition domain; TRAF: TNF-receptor-associated factors-like domain; CD: catalytic domain; UBL: ubiquitin-like domain. (**b**,**c**) GST pull-down assays for DNMT1–USP7 interaction. Recombinant DNMT1 (full-length and truncations) were incubated with GST–USP7 (various truncations) proteins immobilized on glutathione resin. The bound proteins were analysed using SDS–PAGE and Coomassie blue staining. All target proteins in the input are indicated with stars, and bound proteins are indicated with triangles. Molecular weight markers were shown as indicated.

To investigate the interaction between DNMT1 and USP7, we performed glutathione *S*-transferase (GST) pull-down assays using purified full-length and truncations of human DNMT1 and USP7. Tandem UBL domains of USP7 (residues 560–1,102, designated TUD^USP7^), but not the catalytic domain (CD^USP7^) or TRAF domain of USP7, strongly bound to DNMT1 (Fig. 1a,b). Full-length and a truncation (residues 600–1,600), but not the two N-terminal fragments, of DNMT1 bound to TUD^USP7^ (Fig. 1c). Isothermal titration calorimetry measurement shows that DNMT1 (residues 600–1,600) binds to TUD^USP7^ with a binding affinity of 0.60 μM (Supplementary Fig. 1a). Heterodimer formation in solution was confirmed by size exclusion chromatography (Supplementary Fig. 1b). Consistent with a previous observation, USP7 slightly enhanced the *in vitro* DNA methyltransferase activity of DNMT1 by a factor of 2 (Supplementary Fig. 1c)^17^. However, whether this enhancement in activity is physiologically relevant remains unknown.

### Overall structure of DNMT1–USP7

**Figure 2:**
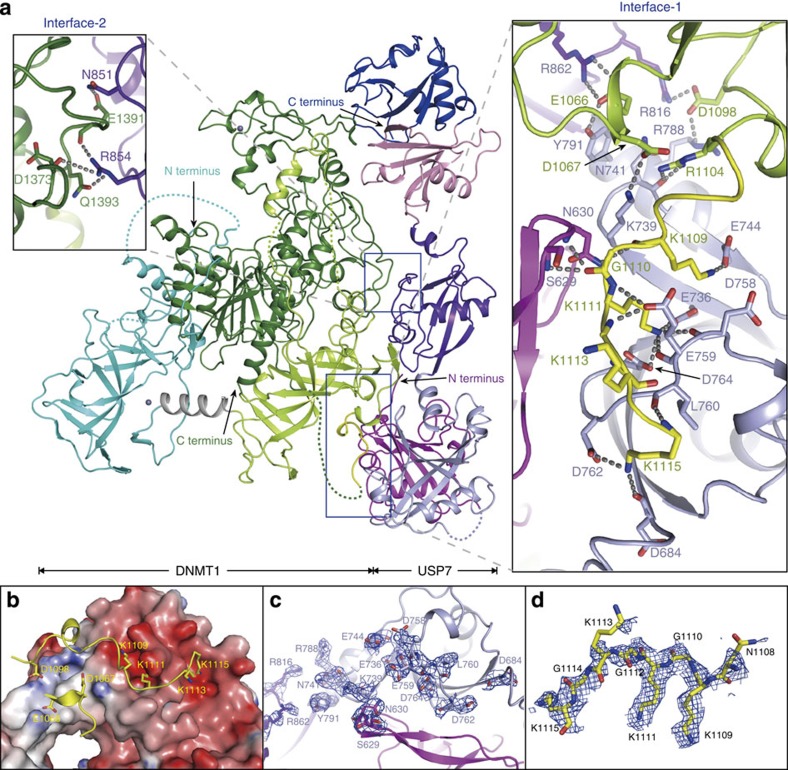
Overall structure of DNMT1 and USP7. (**a**) Ribbon representations of the overall structure of the DNMT1–USP7 complex with the two major interfaces shown in closed-up views. The N- and C-termini of both proteins are indicated, and disconnected regions are shown as dashed lines. Zinc cations are shown as grey balls. Critical residues for the interactions are shown in stick representation. Hydrogen bonds are shown as dashed lines, 3.4 Å was used as the cutoff for the hydrogen bonds in the paper. (**b**) Interactions between KG^DNMT1^ and UBL1–2^USP7^. UBL1–2^USP7^ is shown as an electrostatic potential surface, and KG^DNMT1^ is shown in ribbon representation with critical residues (K1109, K1111 and K1115) shown in stick representation. (**c**,**d**) 2*F*_o_−*F*_c_ omit maps of UBL1–2^USP7^ (**c**) and KG^DNMT1^ (**d**). The maps were calculated at 2.9 Å, contoured at 1.0σ, and are shown as a blue mesh. Most of the critical residues are well-covered by the electron density, indicating that the model was correctly built.

We next determined the crystal structure of DNMT1 (residues 600–1,600, designated as DNMT1 if not specified) in complex with TUD^USP7^ at 2.9 Å resolution (Table 1). In the complex structure, USP7 binds to the BAH2 domain (lemon) and target recognition domain (TRD) of DNMT1 (green) on two separate interfaces (Interface-1 and Interface-2) (Fig. 2a and Supplementary Fig. 2a). USP7 is located at a distant site from the catalytic center of DNMT1. Therefore, its association is unlikely to affect substrate recognition of DNMT1. A structural comparison indicates that DNMT1 in the complex structure adopts a similar fold to that in DNMT1-DNA structure (PDB 3PTA)^27^, with a root-mean-square deviation of 0.97 Å for 802 aligned Cα atoms (Supplementary Fig. 2b).

In the DNMT1–USP7 structure, TUD^USP7^ consist of three separate modules, UBL1–2, UBL3 and UBL4–5, which are connected by two hinge regions flanking UBL3 and collectively adopt an extended conformation (Fig. 2a and Supplementary Fig. 2c). We also compared the structure of TUD^USP7^ (PDB 2YLM) with that in the DNMT1–USP7 complex. Superposition of the UBL1–2 domains from the two structures results in a separation of the respective UBL4–5 domains by as much as 55 Å. Superposition of the UBL4–5 domains from the two structures results in a separation of the respective UBL1–2 domains by as much as 40 Å. This analysis indicates that complex formation leads to a significant conformational change in TUD^USP7^, which primarily occurs at the hinge regions flanking the UBL3 domain. The conformational flexibility of TUD^USP7^ provides a platform for regulation of the enzymatic activity of USP7 (ref. 22).

### The interfaces between DNMT1 and USP7

Two major intermolecular interfaces were observed in the DNMT1–USP7 complex structure (Fig. 2a). Interface-1 primarily involves the KG linker of DNMT1 (residues 1,109–1,119, designated as KG^DNMT1^) and an acidic groove on the surface of UBL1–2^USP7^ (Fig. 2b–d and Supplementary Fig. 2d). The interaction is mediated by a network of hydrogen bonds and salt bridge contacts. Residue K1109^DNMT1^ forms a hydrogen bond with the side chain of residue E744^USP7^ (Fig. 2a). Residue K1111^DNMT1^ snugly inserts into an acidic cave formed by residues E736, D758, E759 and D764 of USP7. Residue K1115^DNMT1^ binds to the carbonyl oxygen atoms of residues D684 and D762 of USP7. The third KG repeat (K1113/G1114) serves as a linker between K1111^DNMT1^ and K1115^DNMT1^ and does not directly interact with USP7. The carbonyl oxygen atoms of residues G1110^DNMT1^ and G1112^DNMT1^ form hydrogen bonds with residues S629, N630 and E759 of USP7. This interaction is further supported by hydrogen bonds formed between residues E1066 and R1104 of DNMT1 and N741, Y791 and R862 of USP7. The Interface-2 involves TRD^DNMT1^ and UBL3^USP7^, with a buried surface area of 676 Å^2^ (Fig. 2a). Residues E1391 and D1373, and Q1393 of DNMT1 form hydrogen bonds with residues N851 and R854 of USP7, respectively. The interactions on the Interface-1 and the Interface-2 are further supported by a number of nonspecific van der Waals contacts. All of the residues involved in the intermolecular interactions are highly conserved, supporting their functional importance (Supplementary Fig. 2e).

### KG linker is essential for the binding of DNMT1 and USP7

**Figure 3:**
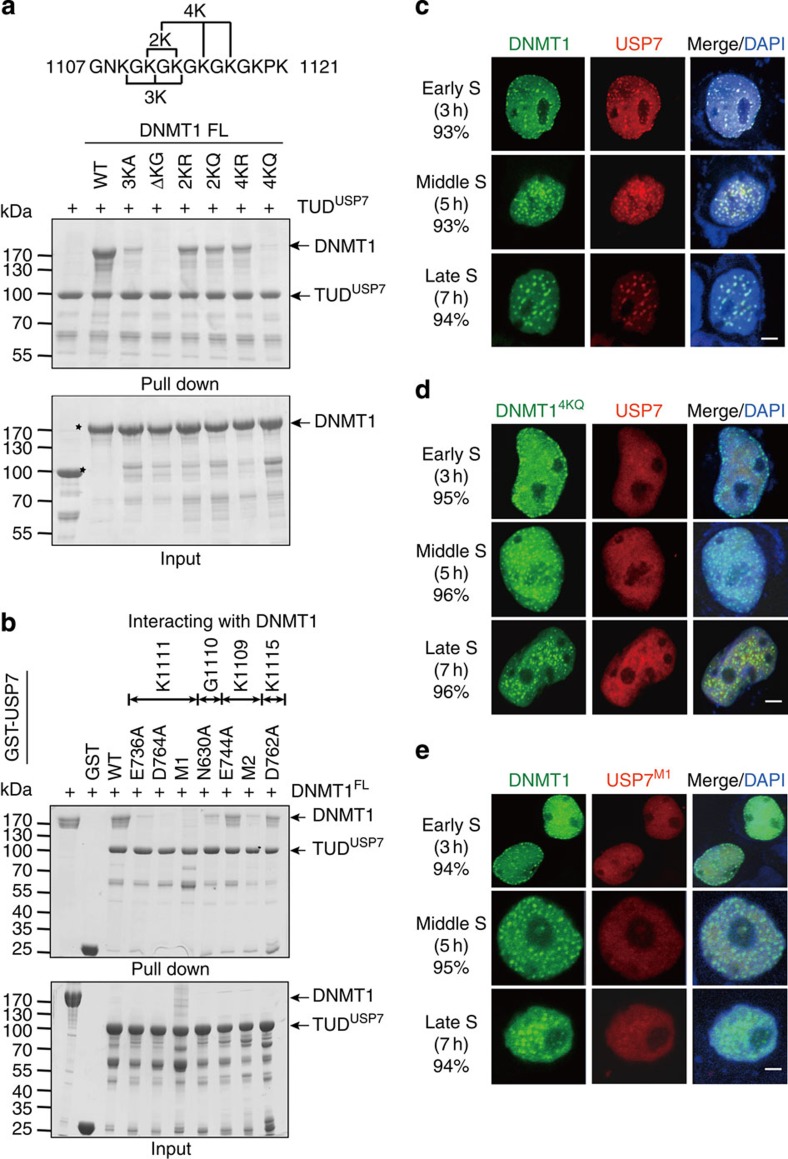
Critical residues for the interaction between DNMT1 and USP7. (**a**,**b**) GST pull-down assays for DNMT1–USP7 interaction. Wild-type and mutant DNMT1 were incubated with wild-type and mutant GST–TUD^USP7^ immobilized on glutathione resin. The bound proteins were analysed using SDS–PAGE and Coomassie blue staining. USP7^M1^: D758A/E759A/D764A; USP7^M2^: N630A/E744A. Molecular weight markers were shown as indicated. (**c**–**e**) Subcellular localization of DNMT1 and USP7 examined by immunofluorescence. GFP–DNMT1 (wild-type and mutants) and RFP–USP7 (wild-type and mutants) were transiently expressed in HEK293T cells followed by double thymidine block. The cells were released at indicated time and used for immunofluorescence assays. DNA was visualized with DAPI staining. The representative staining is shown. The phases of the cell cycle and the percentage of the cells (among 100 cells) with representative staining are indicated. Scale bar, 5 μm.

Previous mass spectrometry analyses have indicated that the four Lysine residues (K1111/K1113/K1115/K1117) of KG^DNMT1^ are acetylated^28^,^29^, suggesting that DNMT1–USP7 interaction might be regulated by acetylation. Lysine to Glutamine (KQ) and Lysine to Arginine (KR) mutations are commonly used to mimic acetylated Lysine and Lysine that is unable to be acetylated, respectively. We therefore mutated two Lysine residues (K1111/K1113) or four Lysine residues (K1111/K1113/K1115/K1117) to Arginine (DNMT1^2KR^ and DNMT1^4KR^) or Glutamine (DNMT1^2KQ^ and DNMT1^4KQ^) to evaluate their effect on DNMT1–USP7 interaction (Fig. 3a). Consistent with above structural analyses, deletion of the KG^DNMT1^ (DNMT1^ΔKG^) and the mutation K1109A/K1111A/K1113A of DNMT1 (DNMT1^3KA^) abolished and largely decreased their interactions with USP7, respectively. DNMT1^2KQ^ slightly decreased the interaction, whereas DNMT1^4KQ^ markedly decreased the binding affinity to USP7, further supporting their importance for DNMT1–USP7 interaction. Intriguingly, DNMT1^2KR^ and DNMT1^4KR^ also slightly decreased the binding affinity to USP7 (Fig. 3a). A possible explanation is that the side chain of Arginine is longer and larger than that of Lysine and may not fit perfectly into this deep acidic pocket in UBL1–2^USP7^.

We also performed the GST pull-down assay using USP7 mutants, in which K1111-interacting residues (D736, D764, D758 and E759), K1109-interacting residues (E744), G1110-interacting residue (N630) and K1115-interacting residue (D762) were mutated to Alanine. As shown in Fig. 3b, mutations D736A^USP7^, D764A^USP7^ and D758A/E759A/D764A (designated as USP7^M1^) abolished DNMT1–USP7 interaction. In addition, mutations N630A^USP7^ and N630A/E744A (designated USP7^M2^) largely decreased the interaction. In contrast, mutations E744A^USP7^ and D762A^USP7^ showed no such effect. The results indicate that K1111-interacting residues on the Interface-1 of USP7 play critical roles in mediating the intermolecular interaction. In contrast, mutation N851A/R854A (USP7^M3^) on the Interface-2 did not decrease the interaction between DNMT1 and USP7 (Supplementary Fig. 3a). Circular dichroism (CD) measurements show that wild-type (WT) and USP7^M1^ proteins have similar secondary structure composition, suggesting that the overall structure of TUD^USP7^ is not disrupted by the mutations (Supplementary Fig. 3b). Taken together, the KG^DNMT1^ (especially residue K1111) and the acidic pocket of the TUD^USP7^ on the Interface-1 are important for the interaction between DNMT1 and USP7.

We next performed immunoprecipitation assays to investigate DNMT1–USP7 interaction in HEK293T cells. Consistent with the observation from GST pull-down assays, DNMT1^3KA^ and DNMT1^ΔKG^ abolished the interaction with USP7, and DNMT1^2KR^, DNMT1^2KQ^, DNMT1^4KR^ and DNMT1^4KQ^ markedly impaired the interaction with USP7 (Supplementary Fig. 3c). Two additional mutants, DNMT1^1KR^ (K1111R) and DNMT1^1KQ^ (K1111Q), also largely impaired DNMT1–USP7 interaction. In addition, mutation E1066A/D1067A (ED-AA) of DNMT1 slightly impaired the interaction, but mutation E1391A/Q1393A (EQ-AA) of DNMT1 showed little effect. Consistently, USP7^M1^, but not USP7^M3^ or USP7^CS^ (mutation C223S of USP7, a catalytically inactive mutant) largely decreased the binding affinity to DNMT1 (Supplementary Fig. 3d). These results indicate that KG^DNMT1^ and UBL1–2^USP7^ on the Interface-1 are essential for DNMT1–USP7 interaction, whereas residues on the Interface-2 are less important.

### DNMT1 recruits USP7 for its chromatin localization

Previous studies indicate that DNMT1 is recruited to DNA replication foci by UHRF1 in the S-phase, and the patterns for its localization vary at different stages of cell cycle^6^,^7^. Cell cycle synchronization was conducted using double thymidine block. The cells were released and collected at the indicated time, and the stages of cell cycle were determined by flow cytometry (Supplementary Fig. 4a). Immunofluorescence assays show that exogenous USP7 primarily localizes in the nucleus and colocalizes with DNMT1 throughout the S-phase in most (∼93%) counted cells (Fig. 3c and Supplementary Fig. 4b). DNMT1^ΔKG^ and DNMT1^4KQ^ exhibited a pattern of cellular localization similar to that of WT DNMT1. However, USP7 was spread throughout the nucleus and did not colocalize with DNMT1 in cells overexpressing DNMT1^ΔKG^ or DNMT1^4KQ^ in most (∼96%) counted cells (Fig. 3d and Supplementary Fig. 4c). Consistently, USP7^M3^, but not USP7^M1^, colocalized with DNMT1 in the S-phase (Fig. 3e and Supplementary Fig. 4d). In support of the above observation, co-immunoprecipitation assays show that DNMT1 binds to USP7, whereas DNMT1^4KQ^ and USP7^M1^ abolished the DNMT1–USP7 interaction across the cell cycle (Supplementary Fig. 4e). These results suggest that DNMT1 binds to USP7 *in vivo* and that DNMT1 localization is independent of USP7, whereas USP7 localizes to chromatin in a manner that is dependent on DNMT1–USP7 interaction.

### DNMT1–USP7 interaction is required for DNMT1 stabilization

**Figure 4:**
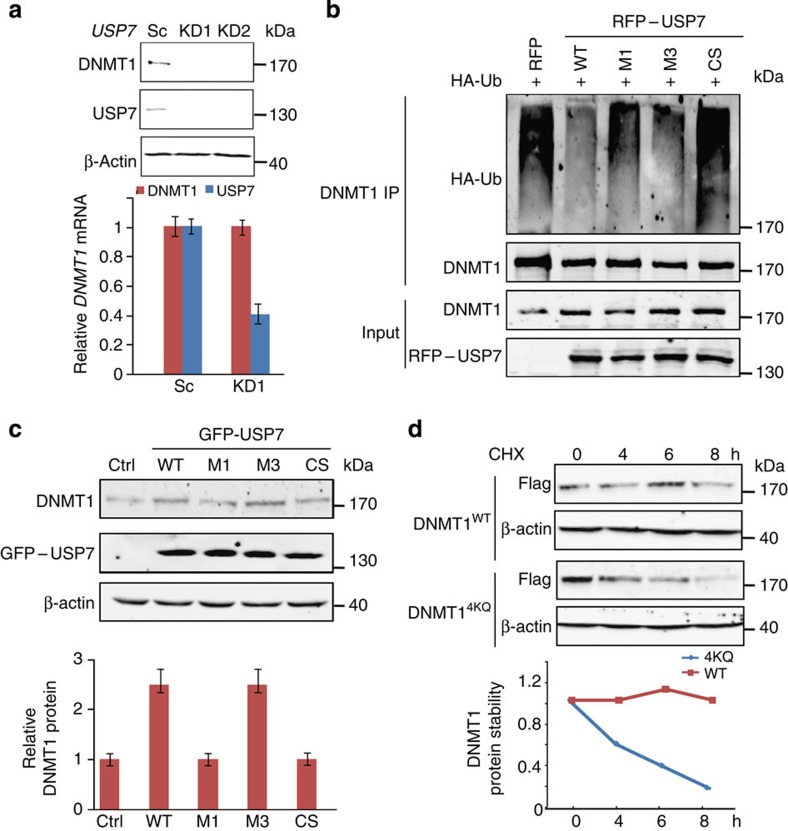
USP7–DNMT1 interaction is required for USP7-mediated stabilization of DNMT1. (**a**) DNMT1 protein levels in HEK293T cells stably expressing USP7 shRNAs. The protein levels were determined by immunoblotting, and the messenger RNA levels were determined by quantitative real-time PCR. The error bars represent ±s.d. from triplicate experiments. Uncropped blots are shown in Supplementary Fig. 7. (**b**) Effect of USP7–DNMT1 interaction on ubiquitination of DNMT1. HEK293T cells were cotransfected with either wild-type or mutant RFP–USP7 and HA–ubiquitin, followed by immunoprecipitation of DNMT1. Polyubiquitination was detected by immunoblotting with HA antibody. As input, the whole cell lysates were analysed by immunoblotting using the indicated antibodies. Uncropped blots are shown in Supplementary Fig. 7. (**c**) Effect of USP7–DNMT1 interaction on DNMT1 protein stability. HEK293T cells were infected with GFP-tagged wild-type and mutant USP7 lentivirus. The protein levels of DNMT1, USP7 and β-actin were determined (top), and the relative protein level of DNMT1 was quantified (bottom). The error bars represent ±s.d. from triplicate experiments. Uncropped blots are shown in Supplementary Fig. 7. (**d**) Protein stabilities of DNMT1 and DNMT1^4KQ^. HEK293T cells stably expressing DNMT1 and DNMT1^4KQ^ were treated with CHX (100 μg ml^−1^) and harvested at the indicated time points. The expression levels of DNMT1 and β-actin were determined (top), and the relative DNMT1 protein level was quantified (bottom). Uncropped blots are shown in Supplementary Fig. 7.

Consistent with previous studies that USP7 deubiquitinates and stabilizes DNMT1 (refs 16, 17, 18), knockdown of *USP7* in several cell lines (HEK293T, HeLa, HCT116 p53−/− and p53+/+ cells) resulted in a significant decrease in the DNMT1 protein level, but not in the *DNMT1* messenger RNA level (Fig. 4a and Supplementary Fig. 5a,b). A similar effect was observed in HCT116 *USP7* knockout cells (Supplementary Fig. 5c). The ubiquitination level of DNMT1 is increased in two independent *USP7* knockdown cells (Supplementary Fig. 5d). USP7^WT^, but not USP7^CS^, largely decreased the ubiquitination level of DNMT1 *in vitro* (Supplementary Fig. 5e). In the *in vivo* deubiquitination assays, USP7^WT^ and USP7^M3^ largely decreased the ubiquitination level of DNMT1, whereas USP7^M1^ and USP7^CS^ showed no such effect (Fig. 4b). The overexpression of USP7^WT^ or USP7^M3^, but not USP7^M1^ or USP7^CS^, markedly increased the protein level of DNMT1 in HEK293T cells (Fig. 4c). In addition, DNMT1^WT^ was stable in HEK293T cells, whereas DNMT1^4KQ^ tended to undergo degradation from 4 h after treatment with cycloheximide (CHX, an inhibitor of protein synthesis) (Fig. 4d). Collectively, USP7 binds to and prevents the protein degradation of DNMT1 in a manner that is dependent on its deubiquitinase activity and its interaction with DNMT1.

### Acetylation of KG linker stimulates degradation of DNMT1

**Figure 5:**
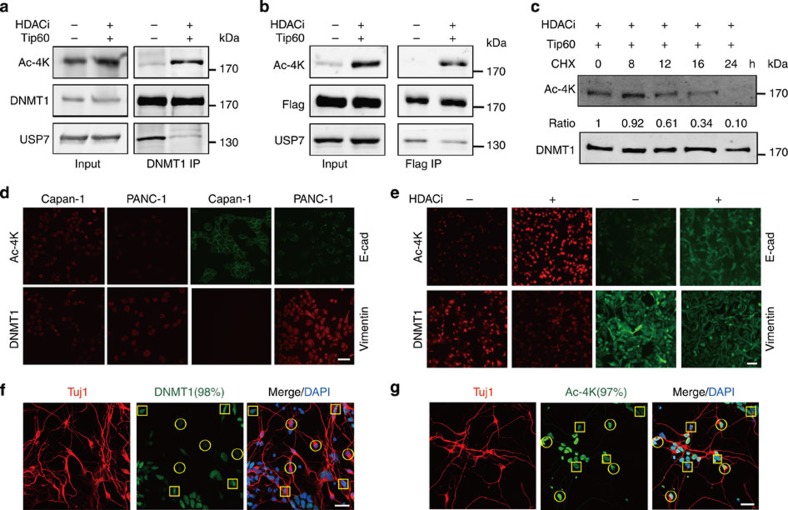
**Acetylation of KG**^**DNMT1**^ **impairs DNMT1–USP7 interaction and promotes the degradation of DNMT1.** (**a**,**b**) Acetylation of KG^DNMT1^ impairs DNMT1–USP7 interaction. HEK293T cells were transfected with Tip60 and treated with HDACi (TSA, MS-275 and NAM). Endogenous (**a**) or exogenous (**b**) DNMT1 was immunoprecipitated with antibodies against DNMT1 and Flag, respectively. The protein levels were analysed by immunoblotting. Uncropped blots are shown in Supplementary Fig. 7. (**c**) Acetyl-DNMT1 is less stable compared with DNMT1. HEK293T cells were transfected with Flag–DNMT1 and Tip60, followed by treatment with HDACi (TSA and MS-275). The cells were treated with CHX (100 μg ml^−1^) and harvested at the indicated time. The relative protein levels of acetyl-DNMT1 were determined and indicated below. Uncropped blots are shown in Supplementary Fig. 7. (**d**) Negative correlation between DNMT1 and acetyl-DNMT1 in PANC-1 (low E-cadherin and high Vimentin) and Capan-1 (low Vimentin and high E-cad) pancreatic cancer cells. Scale bar, 20 μm. (**e**) HDACi (TSA, MS-275 and NAM) treatment increases the protein levels of acetylation of KG^DNMT1^ and E-cad, and decreases the protein level of DNMT1 and Vimentin in PANC-1 cells. Scale bar, 20 μm. (**f**,**g**) Negative correlation between DNMT1 (**f**) and acetyl-DNMT1 (**g**) in differentiated neuronal cells. Neuronal cells were stained with an anti-Tuj1 antibody, and DNA was visualized with DAPI staining. The representative staining is shown and the percentage of the cells (among 100 cells) with representative staining are indicated. Differentiated neuronal cells and non-neuronal cells are indicated in yellow circles and squares, respectively. Scale bar, 20 μm.

We next tested whether acetylation regulates the degradation of DNMT1. On treatment of the cells with CHX, DNMT1 underwent marked degradation in the presence (but not the absence) of histone deacetylase inhibitors (HDACi), including MS-275 and trichostatin (TSA) (Supplementary Fig. 5f). This effect was observed in three different cell lines (HeLa, PC-3 and A549 cells), suggesting a general mechanism for acetylation-regulated degradation of DNMT1. To investigate whether acetylation of KG^DNMT1^ directly regulates DNMT1 protein stability, we raised a polyclonal antibody against the acetylation of four Lysine residues K1111/K1113/K1115/K1117 encompassing the KG^DNMT1^ (designated Ac-4K antibody) (Supplementary Fig. 6a–d). The addition of HDACi and nicotinamide (NAM) and transfection of histone acetyltransferase Tip60 (ref. 16) largely increased the level of acetylated KG^DNMT1^ (designated as acetyl-DNMT1 hereafter) for both endogenous and exogenous DNMT1 and impaired the interaction between DNMT1 and USP7 (Fig. 5a,b). Exogenous DNMT1 was stable following CHX treatment, whereas acetyl-DNMT1 tended to undergo degradation after 12 h under similar experimental conditions, indicating that acetylation of KG^DNMT1^ promotes the degradation of DNMT1 (Fig. 5c). These results support the hypothesis that acetylation of KG^DNMT1^ impairs the otherwise stable interaction between DNMT1 and USP7 and therefore allows protein degradation of DNMT1 to occur. Notably, HDACi treatment had no obvious effect on the level of DNMT1 methylation (K142me1), which was previously shown to regulate DNMT1 stability (Supplementary Fig. 6e)^13^.

### Acetyl-DNMT1 is negatively correlated with DNMT1 *in vivo*

Since acetylation of DNMT1 disrupts the interaction between DNMT1 and USP7, and results in the degradation of DNMT1, we therefore tested whether acetyl-DNMT1 is negatively correlated with DNMT1 *in vivo*. The increased protein level of DNMT1 has been observed during the development of pancreatic cancer from normal tissue to pancreatic ductal adenocarcinoma^30^,^31^. Two pancreatic cancer cell lines, PANC-1 (poorly differentiated, high level of Vimentin and low level of E-cadherin) and Capan-1 (well-differentiated, high level of E-cadherin and low level of Vimentin), were used to investigate the correlation between acetyl-DNMT1 and DNMT1 *in vivo*. Consistent with previous studies^32^,^33^,^34^, immunofluorescence and immunoblotting assays indicate that the protein level of DNMT1 is higher in PANC-1 than that in Capan-1 cells (Fig. 5d and Supplementary Fig. 6f). In contrast, the level of acetyl-DNMT1 is lower in PANC-1 than that in Capan-1 cells. Treatment of PANC-1 cells with HDACi led to a marked increase in the protein level of acetyl-DNMT1 and a decrease in the protein level of DNMT1 (Fig. 5e and Supplementary Fig. 6g). These results indicate a negative correlation between acetyl-DNMT1 and DNMT1 in the two pancreatic cancer cell lines. These results also provide a possible explanation for the enhanced anti-proliferative effect of the combination of HDACi and 5-aza-CdR (a DNMT1 inhibitor) on pancreatic cancer cells^35^ because cancer cells with lower DNMT1 expression are more sensitive to 5-aza-CdR treatment^34^. Negative correlation of the protein levels of acetyl-DNMT1 and DNMT1 was also observed in neuronal cells differentiated from iPS cells. The neuronal cells were stained with Tuj1 antibody^36^. Compared with that in undifferentiated cells, the protein level of DNMT1 decreased in most (∼98%) differentiated normal neuronal cells and the level of acetyl-DNMT1 increased in most (∼97%) differentiated normal neuronal cells (Fig. 5f,g). These results suggest that a decrease in acetyl-DNMT1 restores otherwise impaired interaction between DNMT1 and USP7 and therefore leads to an increase in DNMT1 protein level during the differentiation of neuronal cells.

## Discussion

DNMT1 is an important epigenetic regulator and plays a key role in the maintenance of DNA methylation during DNA replication. Dysregulations of DNMT1 and aberrant DNA methylation are frequently observed in many human cancers and inhibitors for DNMT1 have been used in clinical trials for therapeutic applications. USP7 is the first identified and only known deubiquitinase that binds to and stabilizes DNMT1. In this work, we determined the crystal structure of DNMT1–USP7 complex and revealed an essential interface for the intermolecular interaction between DNMT1 and USP7. Structural and biochemical analyses indicate that the KG linker of DNMT1 is essential for its interaction with USP7, which is indispensible for the stabilization of DNMT1 *in vivo*. An acetylation mimic mutant (DNMT1^4KQ^) or acetylation of four Lysine residues on the KG linker of DNMT1 led to a decrease in its binding affinity to USP7 and a decrease in its protein stability. Negative correlation of acetyl-DNMT1 and DNMT1 was observed in differentiated neuronal cells and pancreatic cancer cells, and decrease of acetyl-DNMT1 was observed in pancreatic ductal adenocarcinoma. HDAC inhibitors treatment increased the level of acetylation of the KG linker of DNMT1 and decreased the level of DNMT1 in the above cell lines. Taken together, our studies reveal the molecular mechanism for USP7-mediated stabilization of DNMT1 regulated by acetylation of KG linker of DNMT1.

Although previous studies show that USP7 binds to and regulates DNMT1 stability^16^,^17^,^18^, the molecular mechanism remains largely unknown. Here we demonstrate that DNMT1 stability is regulated by acetylation on its KG linker (K1111/K1113/K1115/K1117), which results in a dissociation of USP7 from DNMT1 and promotes proteasomal degradation of DNMT1. We did not observe the anti-correlation between DNMT1 and acetyl-DNMT1 during cell cycle in HeLa cells (Supplementary Fig. 6h), suggesting that acetylation may regulate the degradation of DNMT1 under specific physiological or pathological conditions, such as tumorigenesis and development of neuronal disease. Our studies add another layer of regulation of DNMT1 stability and provide structural basis for understanding the molecular mechanism.

DNMT1 inhibitors, 5-aza and 5-aza-cdR, have been used for clinical trials in the treatment of acute myeloid leukaemia and myelodysplastic syndrome^37^,^38^. Combinational use of DNMT1 inhibitor and HDAC inhibitor is more effective on acute myeloid leukaemia and advanced Ewing's sarcoma (compared with single inhibitor used)^39^,^40^. Our results show that HDAC inhibitors treatment increases DNMT1 acetylation and stimulates its degradation. The observation is consistent with previous study that the gene expression patterns are similar in cells treated with DNMT1 inhibitor and HDAC inhibitor^41^. Cells with lower DNMT1 expression are more sensitive to 5-aza-CdR treatment for pancreatic cancers^34^. Thus, our studies also provide a possible explanation for better effect when combination of HDAC inhibitor and DNMT1 inhibitor is used in clinical trials.

DNMT1 is a multidomain-containing protein and is known to interact with a number of proteins, including UHRF1 and PCNA at replication foci, histone modifiers (HDAC1 and EZH2) and transcription factors (DMAP1 and CFP1)^10^,^11^,^12^. UHRF1 is one of the most important DNMT1-interacting partners, which recruits DNMT1 to chromatin and is required for maintenance of DNA methylation^6^,^7^. However, we did not observe stable interaction between UHRF1 and DNMT1 using purified recombinant proteins (data not shown). In contrast, DNMT1 and USP7 form stable complex even in a solution containing 300 mM NaCl (Supplementary Fig. 1a,b), indicating a strong binding affinity between the two proteins. The immunofluorescence assay also showed precise colocalization of DNMT1 and USP7 at the DNA replication foci. These results strongly suggest that USP7 physically and functionally associates with DNMT1 under physiological conditions. Besides DNMT1, USP7 also deubiquitinates other chromatin-associated proteins, including histone H2B, UHRF1 and Tip60 (refs 16, 18, 21, 23, 24). Notably, DNMT1, UHRF1, Tip60 and other two epigenetic regulators (histone deacetylase HDAC1 and histone H3K9 methyltransferase G9a) exist in a macro-molecular complex that is involved in the maintenance of epigenetic information in S-phase of cell cycle^42^. It is tempting to speculate that USP7 might also deubiquitinate and stabilize other proteins in this complex or chromatin-associated proteins yet to be discovered.

The functions of USP7 have been extensively studied because the deubiquitinase regulates protein stability of p53 and MDM2, two critical regulators controlling tumorigenesis^19^. Structural studies indicate that N-terminal TRAF domain of USP7 is responsible for recognition of p53 and MDM2, which bind the same site of TRAF domain, revealing a competitive nature of the interactions^43^. Epstein–Barr nuclear antigen 1 protein of Epstein–Barr virus also binds to the same substrate-binding pocket with a stronger binding affinity than that of p53. In response to latent Epstein–Barr virus infection, USP7 binds to Epstein–Barr nuclear antigen 1 and could not stabilize p53 (ref. 44). The C-terminal tandem UBL domain of USP7 is also known to regulate enzymatic activity of USP7 (ref. 22), and interacts with ICP0 (refs 20, 45) and UHRF1 (ref. 24). Our studies, for the first time, provide three-dimensional structure of a C-terminal substrate-binding site of USP7, which is a deep acidic pocket located in C-terminal tandem UBL domains. This substrate-binding site may prefer basic residues (mainly Lysine) for substrate recognition because it strongly binds to KG linker of DNMT1 and even K-to-R mutation impairs the interaction. It would also be of interest to test whether other proteins, such as ICP0 and UHRF1, also bind to USP7 at the same pocket. Our studies also show that mutation of residues on this pocket of USP7 abolished the binding affinity and deubiquitination activity to DNMT1 and promoted degradation of DNMT1, and therefore provide a structural basis for designing inhibitors to block DNMT1–USP7 interaction for therapeutic applications.

## Methods

### Plasmids and shRNA

DNMT1 and mutants were cloned into pET-28a, pCMV–Flag, pLVX-Puro and pEGFP1-C1 vectors. USP7 and mutants were cloned into pET-28a, pLVX-green fluorescent protein–Puro (GFP–Puro) and pHCRed1-C1 vectors. Ubiquitin was cloned into the pCMV–HA vector. The sequences for USP7 short hairpin RNA (shRNA) are as follows: KD1, 5′- tgtatctattgactgcccttt -3′; and KD2, 5′- cgtggtgtcaaggtgtacta -3′. The following primers were used for real-time PCR: USP7 (forward), 5′- ggaagcgggagatacagatga -3′; USP7 (reverse), 5′- aaggaccgactcactcagtct -3′; DNMT1 (forward), 5′- cctagccccaggattacaagg -3′; DNMT1 (reverse), 5′- actcatccgatttggctctttc -3′; GAPDH (forward), 5′- tgatgacatcaagaggtggtgaag -3′; and GAPDH (reverse), 5′- tccttggaggccatgtgggccat -3′.

### Protein purification

Different constructs of human *DNMT1* were subcloned into a modified pET-28a plasmid and expressed in the *Escherichia coli* strain Rosetta (DE3). Human *USP7* and its constructs were subcloned into a modified pGEX-6P-1 plasmid and expressed in the *E. coli* strain BL21(DE3). The proteins were initially purified using Ni-NTA affinity chromatography, followed by on-column cleavage at 4 °C overnight. The proteins were further purified using anion exchange and size exclusion chromatography. To assemble the complex, purified DNMT1 (residues 600–1,600) and USP7 (residues 560–1,102) proteins were mixed in a 1:1 molar ratio, followed by size exclusion chromatography in a buffer containing 20 mM Tris-HCl (pH 8.0), 300 mM NaCl and 5 mM dithiothreitol. The peak fractions were collected and concentrated to 10 mg ml^−1^ for crystallization and biochemical analyses.

### Crystallization and data collection

Crystals of the DNMT1–USP7 complex were grown at 18 °C using the hanging drop vapour diffusion method by mixing equal volumes of the purified protein complex and crystallization buffer containing 6–8% polyethylene glycol 3350, 200 mM potassium acetate or sodium formate, and 100 mM Bis-Tris (pH 6.0–7.0). Crystals were equilibrated with a cryoprotectant buffer containing reservoir buffer and additional 20% glycerol (v/v) and were flash frozen in a cold nitrogen stream at −173 °C. All data were collected in 1.0000 Å wavelength at beamline BL17U at SSRF (Shanghai Synchrotron Radiation Facility, China). The data were processed using the programme HKL2000 (ref. 46).

### Structure determination

The phase was determined by molecular replacement using the programme PHASER^47^ with the crystal structures of DNMT1 (residues 600–1,600; PDB 3SWR) and USP7 (residues 560–1,084; PDB 2YLM) used as search models. The final model was manually built using Coot^48^. All refinements were performed using the refinement module phenix.refine of the PHENIX package^49^. The model quality was validated using the PROCHECK^50^ programme, which indicated good stereochemistry according to the Ramachandran plot for the structure. All structural figures were generated using PyMOL^51^.

### GST pull-down assays

Various truncations of GST-tagged USP7 proteins were incubated with DNMT1 proteins in binding buffer containing 25 mM HEPES (pH 7.4), 150 mM NaCl, 5% glycerol, 0.05% Triton X-100 and 1 mM dithiothreitol for 2 h at 4 °C. The protein samples were then immobilized on 25 μl of glutathione resin (GE Healthcare) for 20 min at 4 °C. The resin was washed three times with binding buffer, and bound proteins were subjected to SDS–PAGE and stained with Coomassie brilliant blue.

### Isothermal titration calorimetry

To obtain the binding affinity between USP7 and DNMT1, 0.05 mM WT DNMT1 (residues 600–1,600) was titrated with 0.5 mM USP7 (560–1,102) using iTC200 microcalorimeter (GE Healthcare) at 18 °C. Both proteins were prepared in a buffer containing 10 mM HEPES (pH 8.0) and 300 mM NaCl. The data were fitted by software Origin 7.0.

### CD spectroscopy

The CD spectra were measured in a 0.1 cm quartz cell using J-715 Circular Dichroism spectropolarimeter (JASCO, Japan). The measurements were carried out in PBS buffer (pH 7.4). The concentration of the samples was 0.2 mg ml^−1^. Two CD spectra from 190 to 250 nm were averaged for each point and sample.

### *In vitro* DNA methyltransferase activity assay

The enzymatic activities of DNMT1 proteins were assessed by incorporation of ^3^H-labelled methyl group from S-adenosyl-l-[methyl-^3^H]methionine ([methyl-^3^H]AdoMet, PerkinElmer). The substrate used in this assay was 14-bp hemi-methylated dsDNA (5′- GGAGGCXGCCTGCT -3′; in the top strand, *X*=5 mC, whereas in the bottom strand *X*=C, with a 5′-biotin label for purification). DNMT1 (0.3 μM) and USP7 (3 μM) proteins were mixed and incubated on ice for 15 min. The biotinylated DNA (2 μM) was methylated by DNMT1 proteins in the presence of 2.5 μM [methyl-^3^H]AdoMet, 25 mM Tris-HCl (pH 7.5) and 1 mg ml^−1^ BSA. The reactions were incubated at 37 °C for 30 min and were terminated with the addition of cold wash buffer (500 mM NaCl and 1 mM EDTA in PBST). The DNA products were immobilized on streptavidin beads, washed three times and subjected to liquid-scintillation counting (PerkinElmer). Each reaction was performed in triplicate.

### Cell culture, transfection and treatment

HEK293T (293T), HeLa, HCT116, PC-3 and A549 cells were obtained from the American Type Culture Collection. Capan-1 and PANC-1 cells were provided by Dr Yingyi Li at Fudan University Shanghai Cancer Center. HEK293T, HeLa and PC-3 cells were maintained in Dulbecco's modified Eagle's medium (Sigma) containing 10% foetal bovine serum (FBS; Biological Industries, Israel) and supplemented with 2 mM Glutamine, 100 U ml^−1^ penicillin and 100 μg ml^−1^ streptomycin. HCT116 cells were maintained in McCoy 5A plus 10% FBS. A549, Capan-1 and PANC-1 were maintained in 1,640 medium containing 10% FBS. Plasmid transfection was performed using linear polyethylenimines (Polysciences).

HDAC inhibitors were purchased from Selleck Chemicals (MS-275), Cell Signaling Technology (TSA) and Sigma-Aldrich (NAM). For HDACi treatment, cells were treated with MS-275 and TSA for 20 h, followed by 4–6 h of NAM treatment prior to collection or followed by CHX treatment with the indicated time for protein degradation.

HEK293T cells were synchronized by double thymidine block and released at indicated time. The stages of cell cycle were determined by flow cytometry. The cells were fixed in 70% ethanol at −20 °C overnight followed by washing with PBS twice. Cells were stained in PBS buffer containing 0.1% Triton X-100, 25 μg ml^−1^ propidium iodide and 50 μg ml^−1^ RNAse for 20 min at 37 °C. Then sample were subject to flow cytometry assay. The synchronized cells were used for immunofluorescence and immunoprecipitation assays.

### Immunoprecipitation

The HEK293T cells were transfected with WT or mutant Flag–tagged DNMT1, and cells were harvested 48 h after transfection. Cells were lysed in RIPA (radioimmunoprecipitation assay) buffer containing a complete protease inhibitor mixture (plus HDAC inhibitors for samples detecting acetylated DNMT1) and incubated with anti–Flag M2 beads (Sigma-Aldrich) for 2 h at 4 °C. The beads were washed five times with washing buffer consisting of 20 mM Tris-HCl (pH 7.9), 300 mM NaCl, 5% glycerol, 0.1% NP-40 and 0.2 mM EDTA. The bound proteins were used for assays or immunoblotting.

### Generation of the Ac-4K antibody

To understand whether acetylation of the KG linker directly regulates DNMT1 protein stability, we raised polyclonal antibody against the acetylation of four Lysine residues K1111/K1113/K1115/K1117 of KG^DNMT1^ using the acetylated peptide corresponding to residues CKGK(ac)GK(ac)GK(ac)GK(ac)GK of DNMT1. The antibody was purified from rabbit serum. The antibody was verified as indicated in Supplementary Fig. 6.

### Immunoblotting

Protein samples were boiled in SDS–PAGE loading buffer and subjected to SDS–PAGE. Proteins were transferred to a nitrocellulose membrane, and the nitrocellulose membrane (Millipore) was blocked using 5% milk in TBST (10 mM Tris-HCl (pH 7.4) 150 mM NaCl and 0.1% Tween 20) buffer at room temperature for 1 h, followed by incubation with the following antibodies: DNMT1(a5495, ABclonal Technology, China), Ac-4K (generated using acetylated DNMT1 peptide as an antigen) and Cyclin B (a2056) from ABclonal Technology, China; USP7 (H-200, Santa Cruz Biotechnology), ubiquitin (3936P, Cell Signaling Technology), HA (sc7392, Santa Cruz Biotechnology), Flag (M20008, Abmart), me-142K-DNMT1 (61497, Active Motif), E-Cad (ab76055, Abcam), Vimentin(V6630, Sigma-Aldrich) and β-actin (M20010, Abmart) at 1:1,000 dilution at 4 °C overnight. Membranes were washed three times with TBST buffer, incubated with horseradish peroxidase (HRP)-conjugated goat anti-rabbit or anti-mouse antibody (Abmart) and visualized on a Tanon-5200 Chemiluminescent Imaging System (Tanon Science & Technology Co., Ltd.).

### Immunofluorescence

For the immunofluorescence assay for DNMT1 and USP7 in HEK293T cells, the cells were transfected with WT or mutant GFP–DNMT1 and red fluorescent protein–USP7 (RFP–USP7) and harvested 48 h after transfection. Cells were fixed with 4% paraformaldehyde for 20 min and washed three times with PBS. Cover slips were mounted with Antifade reagent containing DAPI (Molecular Probes) on slides and examined using a Zeiss LSM 710 microscope.

For immunofluorescence staining of cultured pancreatic tumour cell lines, cells were seeded and allowed to attach overnight. Adherent cells were fixed with 4% paraformaldehyde for 20 min and blocked with 2% BSA and 0.5% Triton X-100 in PBS, followed by incubation with antibodies against DNMT1 (a5495), E-cadherin (ab76055), Vimentin (V6630) and the Ac-4K (generated using acetylated DNMT1 peptide as an antigen) at 1:500 dilution overnight at 4 °C. Cells were subsequently washed three times with PBS and incubated with secondary antibodies containing DAPI (1:1,000 dilution, Invitrogen), as previously described.

A similar procedure was used for the immunofluorescence assay for differentiated neuronal cells. An anti-Beta III Tubulin (Tuj1) (T8578, Sigma-Aldrich) antibody at 1:1,000 dilution was used as a neuronal cell marker.

### *In vitro* deubiquitination assay

Poly-ub-DNMT1 was purified from HEK293T cells cotransfected with Flag–DNMT1 and HA–Ub using anti–Flag M2 beads (Sigma-Aldrich) and eluted with the Flag peptide. The purified Poly-ub-DNMT1 protein was incubated with purified USP7 WT and CS in deubiquitination buffer (50 mM Tris-HCl (pH 8.0) and 0.5 mM EDTA) at 37 °C for 1 h. The reactions were terminated by the addition of SDS loading buffer and subjected to immunoblotting analysis using antibodies against DNMT1 and ubiquitin.

### *In vivo* ubiquitination assay

HEK293T cells were transfected with (Hemagglutinin) HA–ubiquitin and RFP–USP7 (WT and mutants). After 48 h, cells were washed twice with PBS and lysed with RIPA buffer, followed by incubation with protein A agarose preincubated with an anti-DNMT1 antibody at 4 °C overnight. After washing three times with washing buffer, the samples were subjected to immunoblotting analysis using antibodies against DNMT1 and HA.

### Stable cells establishment

Packaging plasmids psPAX2 and pMD2G and lentiviral DNA constructs (pLVX–GFP–Puro for USP7 and mutants, pLVX–Flag for DNMT1 and mutant and pLKO.1 for *USP7* shRNA) were transfected into 293 T cells. Lentiviruses were collected at 48 h after transfection to infect target cells supplemented with 10 μg ml^−1^ polybrene. After 24 h of infection, infected cells were selected by antibiotic to get the stable transgenic cells.

### Generation of differentiated neuronal cells

Epithelial cells were obtained from a healthy individual and were induced to iPS cells using four genes, Oct4, Sox2, c-Myc and Klf4 (ref. 52). Human iPS cells were differentiated to neuronal cells in a neural induction medium using the small molecular agonist purmorphamine (0.1–1.5 μM, Calbiochem, San Diego, CA, USA) for 35 days^36^.

## Author contributions

J.C., H.Y. and Y.X. designed the experiments. J.C. and J.F. performed the protein purification and crystallization of DNMT1–USP7 complex, J.C. collected the data and determined the crystal structure. H.Y. performed all the cell-based assays. R.G. and P.W. performed the protein purification. H.Y. and L.M performed the immunofluorescence of differentiated normal neuronal cells. Z.L. helped in the data analysis. J.C., H.Y. and Y.X. analysed the data and wrote the manuscript. Y.X. supervised the project.

## Additional information

**Accession codes:** The coordinate and structure factor for the DNMT1–USP7 complex structure have been deposited in the Protein Data Bank under accession code 4YOC.

**How to cite this article:** Cheng, J. *et al*. Molecular mechanism for USP7-mediated DNMT1 stabilization by acetylation. *Nat. Commun.* 6:7023 doi: 10.1038/ncomms8023 (2015).

## Supplementary Material

Supplementary Information Supplementary Figures 1-7

## Figures and Tables

**Table 1:** Data collection and refinement statistics.

	**DNMT1–USP7**
*Data collection*	
Space group	*P2*_*1*_*2*_*1*_*2*_*1*_
Cell dimensions	
*a*, *b*, *c* (Å)	110.23, 111.62, 163.87
*α, β, γ* (°)	90, 90, 90
Resolution (Å)	50.0–2.9 (3.0–2.9)*
*R*_sym_ or *R*_merge_	14.6 (85.8)
*I*/*σI*	14.02 (3.86)
Completeness (%)	99.19 (92.23)
Redundancy	13.8 (13.5)
	
*Refinement*	
Resolution (Å)	50.0–2.9 (3.0–2.9)
No. of reflections	44396
*R*_work_/*R*_free_	20.62/25.64
No. of atoms	11386
Protein	11373
Ligand/ion	2
Water	11
*B*-factors	
Protein	66.30
Ligand/ion	67.80
Water	42.50
R.m.s. deviations	
Bond lengths (Å)	0.003
Bond angles (°)	0.89

R.m.s., root-mean-square.

Data set are collected with one native crystal.

^*^Values in parentheses are for highest-resolution shell.
