# Supplementary material for: Molecular mechanism for USP7-mediated DNMT1 stabilization by acetylation
Source: Nat Commun. 2015 May 11;6:7023. doi: 10.1038/ncomms8023 (PMC4432644; doi:10.1038/ncomms8023)
Supplement: Supplementary Information — Supplementary Figures 1-7 [file ncomms8023-s1.pdf]

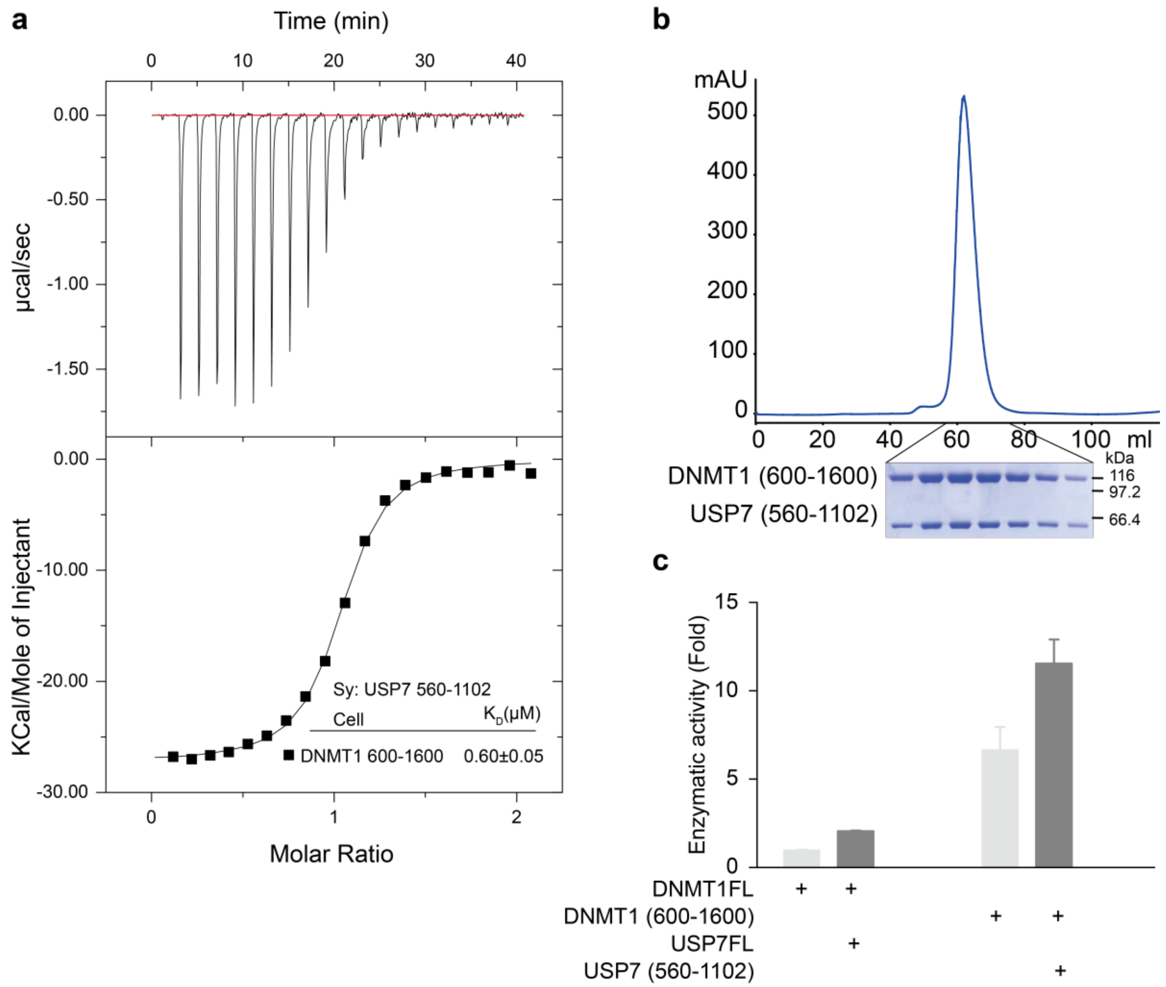

Supplementary Figure 1. Interactions between DNMT1 and USP7

(a) ITC enthalpy plot for the interaction between DNMT1 (residues 600-1600) (in the cell) to USP7 (residues 560-1102) (in the syringe) with the estimated binding affinity (K_D) listed. Sy: syringe.

(b) Size exclusion chromatogram of the human DNMT1 (residues 600-1600) and human USP7 (residues 560-1102) complex using a Superdex 200 column (16/600, GE healthcare) with buffer containing 300 mM NaCl. The peak position is approximately 65 ml, which corresponds to a DNMT1-USP7 heterodimer with a molecular weight of

approximately 180 kDa. The peak fractions were subjected to SDS-PAGE and stained with Coomassie blue.

(c) USP7 enhances the DNA methyltransferase activity of DNMT1 by a factor of two. Enzymatic activities of full-length and truncated DNMT1 were determined in the absence or presence of full-length USP7. Relative enzymatic activities were calculated according to that of full-length DNMT1. The error bars represent ±s.d. from triplicate experiments.

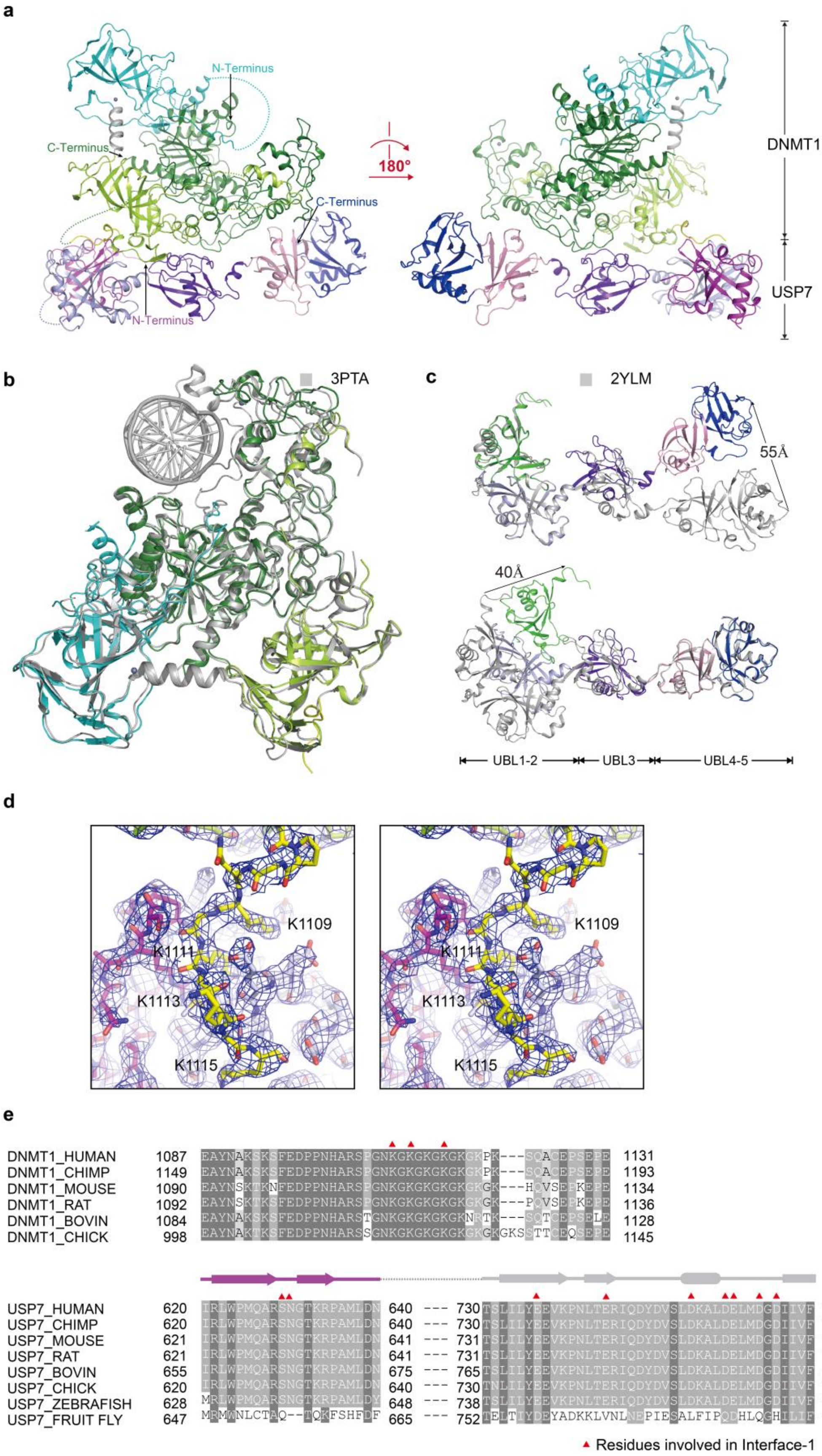
a
N-Terminus
C-Terminus
C-Terminus
N-Terminus
180°
DNMT1
USP7
b
3PTA
c
2YLM
55Å
40Å
UBL1-2
UBL3
UBL4-5
d
K1109
K1111
K1113
K1115
K1109
K1111
K1113
K1115
e
DNMT1_HUMAN 1087 EAYNAKSKSFEDPPNHARSPGNKGKGKGKGKGKPK---SQACEPSEPE 1131
DNMT1_CHIMP 1149 EAYNAKSKSFEDPPNHARSPGNKGKGKGKGKGKPK---SQACEPSEPE 1193
DNMT1_MOUSE 1090 EAYNSKTKNFEDPPNHARSPGNKGKGKGKGKGKGK---HQVSEPKEPE 1134
DNMT1_RAT 1092 EAYNSKTKSFEDPPNHARSPGNKGKGKGKGKGKGK---PQVSEPKEPE 1136
DNMT1_BOVIN 1084 EAYNAKSKSFEDPPNHARSTGNKGKGKGKGKNRTK---SQTCEPSELE 1128
DNMT1_CHICK 998 EAYNAKTKSFEDPPNHARSSGNKGKGKGKGKGKGKGKSSTTCEQSEPE 1145
USP7_HUMAN 620 IRLWPMQARSNGTKRPAMLDN 640 --- 730 TSLILYEEVKPNLTERIQDYDVSLDKALDELMDGDIIVF 768
USP7_CHIMP 620 IRLWPMQARSNGTKRPAMLDN 640 --- 730 TSLILYEEVKPNLTERIQDYDVSLDKALDELMDGDIIVF 768
USP7_MOUSE 621 IRLWPMQARSNGTKRPAMLDN 641 --- 731 TSLILYEEVKPNLTERIQDYDVSLDKALDELMDGDIIVF 769
USP7_RAT 621 IRLWPMQARSNGTKRPAMLDN 641 --- 731 TSLILYEEVKPNLTERIQDYDVSLDKALDELMDGDIIVF 769
USP7_BOVIN 655 IRLWPMQARSNGTKRPAMLDN 675 --- 765 TSLILYEEVKPNLTERIQDYDVSLDKALDELMDGDIIVF 803
USP7_CHICK 620 IRLWPMQARSNGTKRPAMLDN 640 --- 730 TNLILYEEVKPNLTERIQDYDVSLDKALDELMDGDIIVF 768
USP7_ZEBRAFISH 628 MRLWPMQARSNGTKRPAMLDY 648 --- 738 TSLILYEEVKPNLTERIQDYDVSLDKALDELMDGDIIVF 776
USP7_FRUIT FLY 647 MRMWNLCTAQ--TQKFSHFDF 665 --- 752 TELTIYDEYADKKLVNLNEPIESALFIPQDHLQGHILIF 790
Residues involved in Interface-1

Supplementary Figure 2. Structural comparison of DNMT1-USP7 with individual structures

(a) Two different views of the DNMT1-USP7 complex structure shown in ribbon representation. Residues 600-614, 635-702, 855-859, 955-962, 980-983, and 1116-1132 of DNMT1 and 668-674 and 1084-1102 of USP7 are not included in the model due to absence of electron density, which may result from flexibility in the crystals. The N- and C-termini are indicated, and disconnected regions are shown as dashed lines.

(b) Superposition of the DNMT1-USP7 and DNMT1-DNA (PDB ID: 3PTA) structures. USP7 is omitted for clarity. DNMT1 in DNMT1-USP7 is colored as in Figure 2a, and DNMT1 in DNMT1-DNA is colored in gray.

(c) Superposition of UBL domains in the structure of USP7 alone (gray; PDB ID: 2YLM) and in complex with DNMT1 using the UBL1-2^{USP7} (top panel) or UBL4-5^{USP7} (bottom panel) domains. UBL1-2 domains from the two structures superpose well (top panel) with an RMSD of 0.408 Å for 188 Cα atoms. UBL4-5 domains from the two structures superpose well (bottom panel) with an RMSD of 0.523 Å for 176 Cα atoms.

(d) A stereo image of the 2Fo-Fc map for the interaction between KG linker and USP7 in the DNMT1-USP7 structure at 2.9 Å contoured at 1.0σ.

(e) Sequence alignment of critical regions in DNMT1 (top) and USP7 (bottom) that are involved in interactions on the Interface-1. Identical and highly conserved residues are highlighted in dark background and conserved residues in grey background. Secondary structural elements are indicated above the sequences. Critical residues for the interactions are indicated in red triangles.

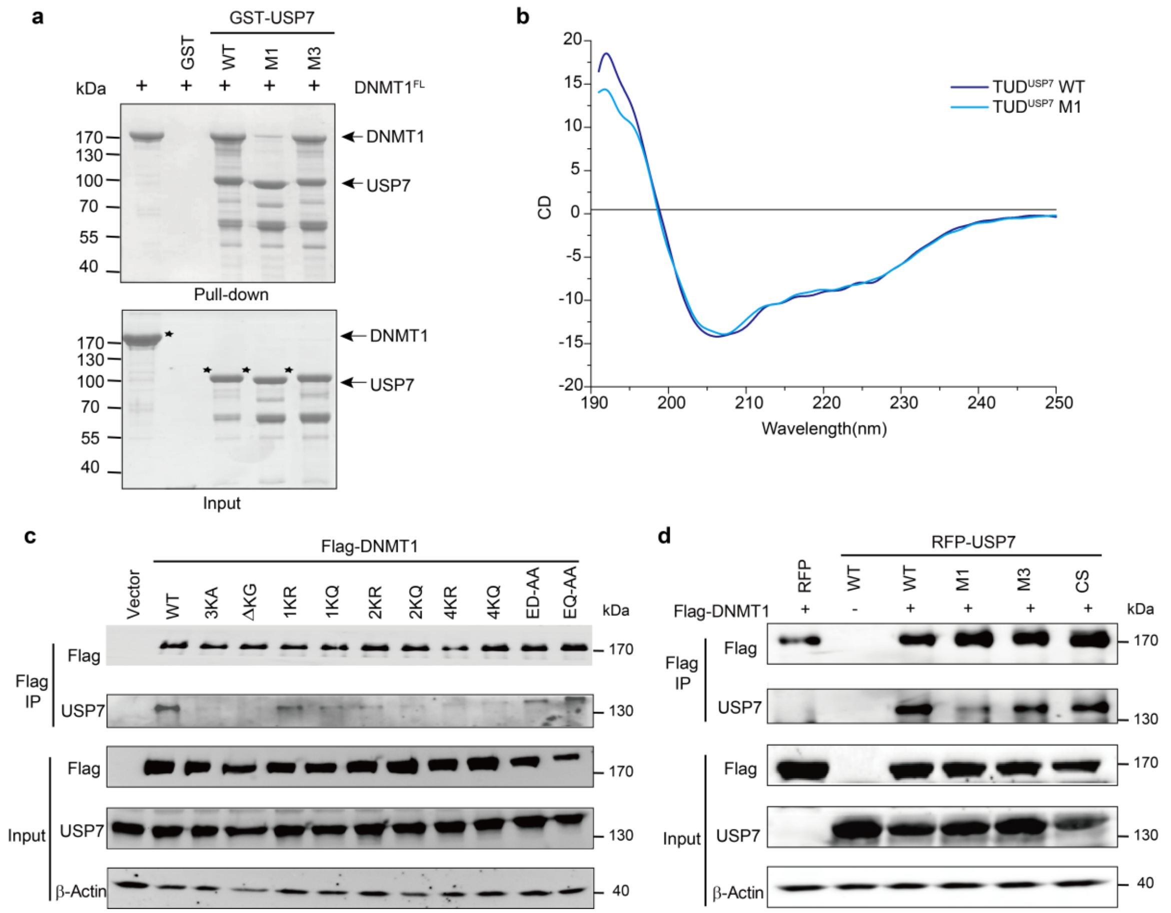

Supplementary Figure 3. ***In vitro*** **and** ***in vivo*** **interactions between DNMT1 and USP7**

(a) GST pull-down assay for DNMT1-USP7 interaction. Wild-type (WT) and two mutants of GST-TUD^{USP7} was immobilized on glutathione resin. The bound DNMT1 full-length protein was analyzed using SDS-PAGE and Coomassie blue staining. Molecular weight markers were shown as indicated. $USP7^{M1}$: D758A/E759A/D764A; $USP7^{M3}$: N851A/R854A.

(b) Circular dichroism (CD) spectra of the wild-type and M1 of TUD^{USP7}. The curves corresponding to the two proteins are colored in blue and cyan, respectively.

(c) HEK293T cells were transiently transfected with Flag-DNMT1 (wild-type and mutants) followed by immunoprecipitation. The proteins were detected by

immunoblotting using the indicated antibodies. As input, the whole cell lysates were analyzed by immunoblotting using the indicated antibodies. Uncropped blots are shown in Supplementary Fig. 7.

(d) HEK293T cells were transiently transfected with RFP-USP7 (wild-type and mutants) and Flag-DNMT1. The interactions were detected as in Supplementary Figure 3c. As input, the whole cell lysates were analyzed by immunoblotting using the indicated antibodies. Uncropped blots are shown in Supplementary Fig. 7.

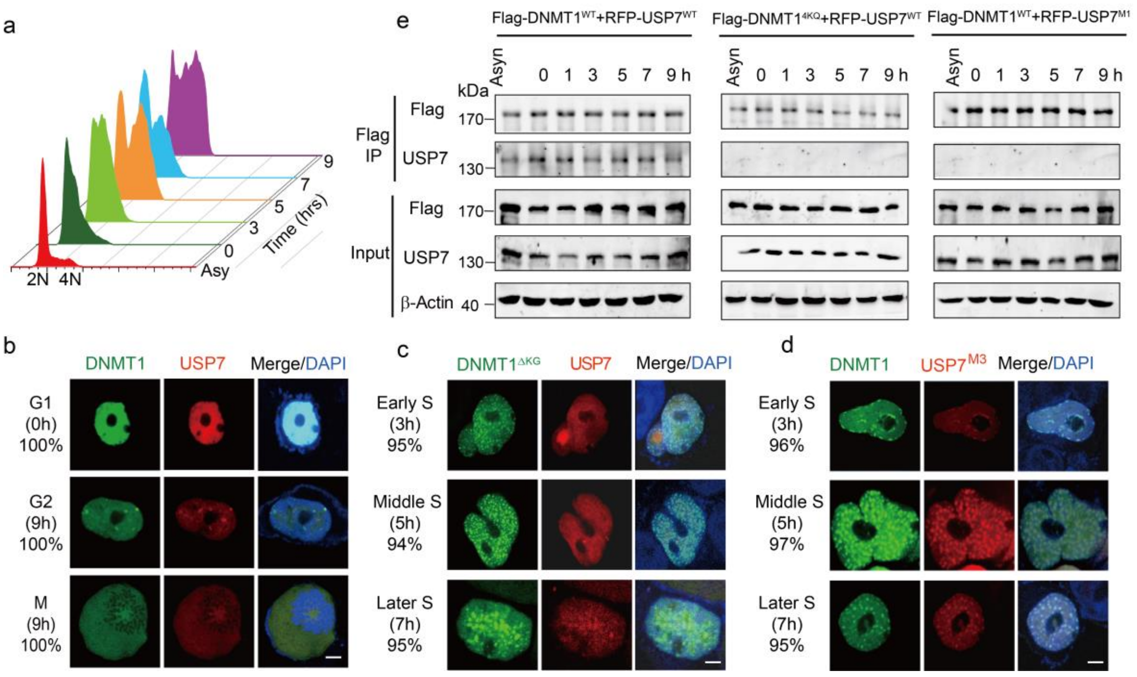

Supplementary Figure 4. DNMT1 and USP7 WT and mutants binding in different stages of the cell cycle.

(a) Flow cytometry profiles of propidium iodide staining of HEK293T cells. The cells were collected at indicated time after double thymidine block. Cells were progressed from G1, S to G2/M phase.

(b) Subcellular localization of DNMT1, USP7 in the G1, G2, and M phases of the cell cycle after double thymidine block. DNA was visualized with DAPI staining. Both proteins localize in the nucleus and dissociate from chromatin in the M phase. Scale bar, 5μm.

(c-d) Subcellular localization of DNMT1 and USP7 examined by immunofluorescence. GFP-DNMT1 (wild-type and mutants) and RFP-USP7 (wild-type and mutants) were transiently expressed in HEK293T cells followed by double thymidine block. The cells were released at indicated time and used for immunofluorescence assays. DNA was visualized with DAPI staining. The representative staining is shown. The phases of the

cell cycle and the percentage of the cells (among 100 cells) with representative staining are indicated. Scale bar, 5μm.

(e) HEK293T cells were transiently transfected with Flag-DNMT1/RFP-USP7, Flag-DNMT1^{4KQ}/RFP-USP7, Flag-DNMT1/RFP-USP7^{M1} and collected at indicated time after double-thymidine block, followed by immunoprecipitation. The proteins were detected by immunoblotting using the indicated antibodies. As input, the whole cell lysates were analyzed by immunoblotting using the indicated antibodies. Uncropped blots are shown in Supplementary Fig. 7.

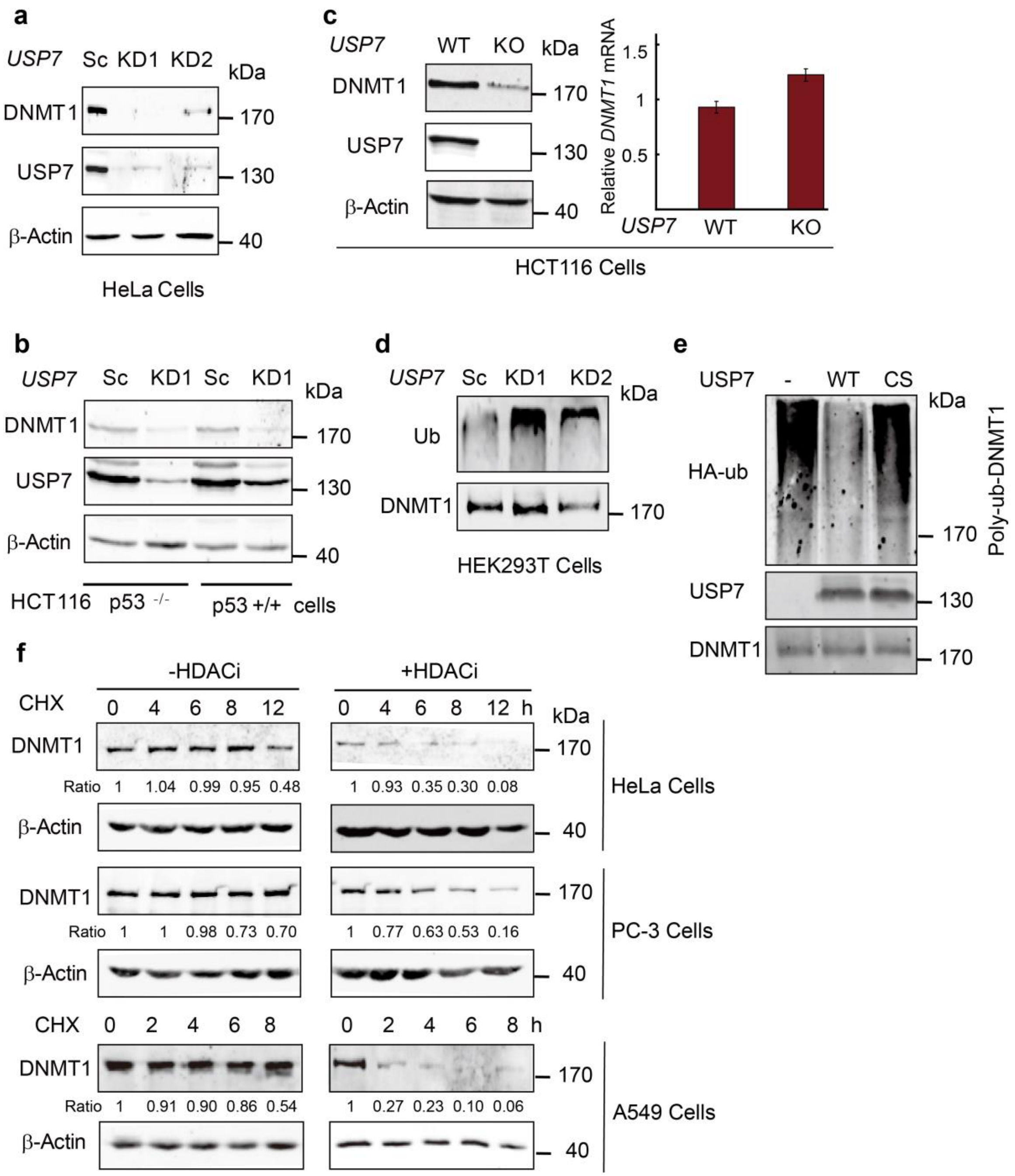

Supplementary Figure 5. USP7 deubiquitylates and stabilizes DNMT1

(a-b) DNMT1 and USP7 protein levels in HeLa cells (a), HCT116 p53 (-/-) and p53 (+/+) cells (b) with knockdown of *USP7*. HeLa, HCT116 p53 (-/-) and p53 (+/+) cells were

infected with scramble or lentivirus expressing two independent USP7 shRNAs. Uncropped blots are shown in Supplementary Fig. 7.

(c) DNMT1 protein and mRNA levels in HCT116 cells with knockout of *USP7*. The HCT116 *USP7* knockout cells were purchased from the Genetic Resources Core Facility of Johns Hopkins University. The protein levels were determined by immunoblotting using the indicated antibodies. The mRNA levels of DNMT1 in wild-type and *USP7* knockout HCT116 cells were determined using qRT-PCR. The error bars represent ±s.d. from triplicate experiments. Uncropped blots are shown in Supplementary Fig. 7.

(d) Endogenous ubiquitination of DNMT1 regulated by USP7. HEK293T cells were infected with two independent USP7 shRNAs. DNMT1 was immunoprecipitated with DNMT1 antibody, followed by immunoblotting using the indicated antibodies. Uncropped blots are shown in Supplementary Fig. 7.

(e) *In vitro* deubiquitination of DNMT1. Flag-DNMT1 was immunoprecipitated with Anti-Flag M2 agarose and eluted with Flag peptide, followed by an *in vitro* deubiquitination assay using recombinant wild-type and $USP7^{CS}$ proteins. The products were subjected to immunoblotting using the indicated antibodies. Uncropped blots are shown in Supplementary Fig. 7.

(f) HeLa, PC-3, and A549 cells were treated with vehicle or HDAC inhibitors (TSA and MS-275) for 20 hours. The cells were further treated with 100 μg/ml CHX and harvested at the indicated time. The cell lysates were subjected to immunoblotting using the indicated antibodies. β-Actin serves as a loading control. The relative protein levels of DNMT1 were determined (compared with that of β-actin), as indicated below each panel. Uncropped blots are shown in Supplementary Fig. 7.

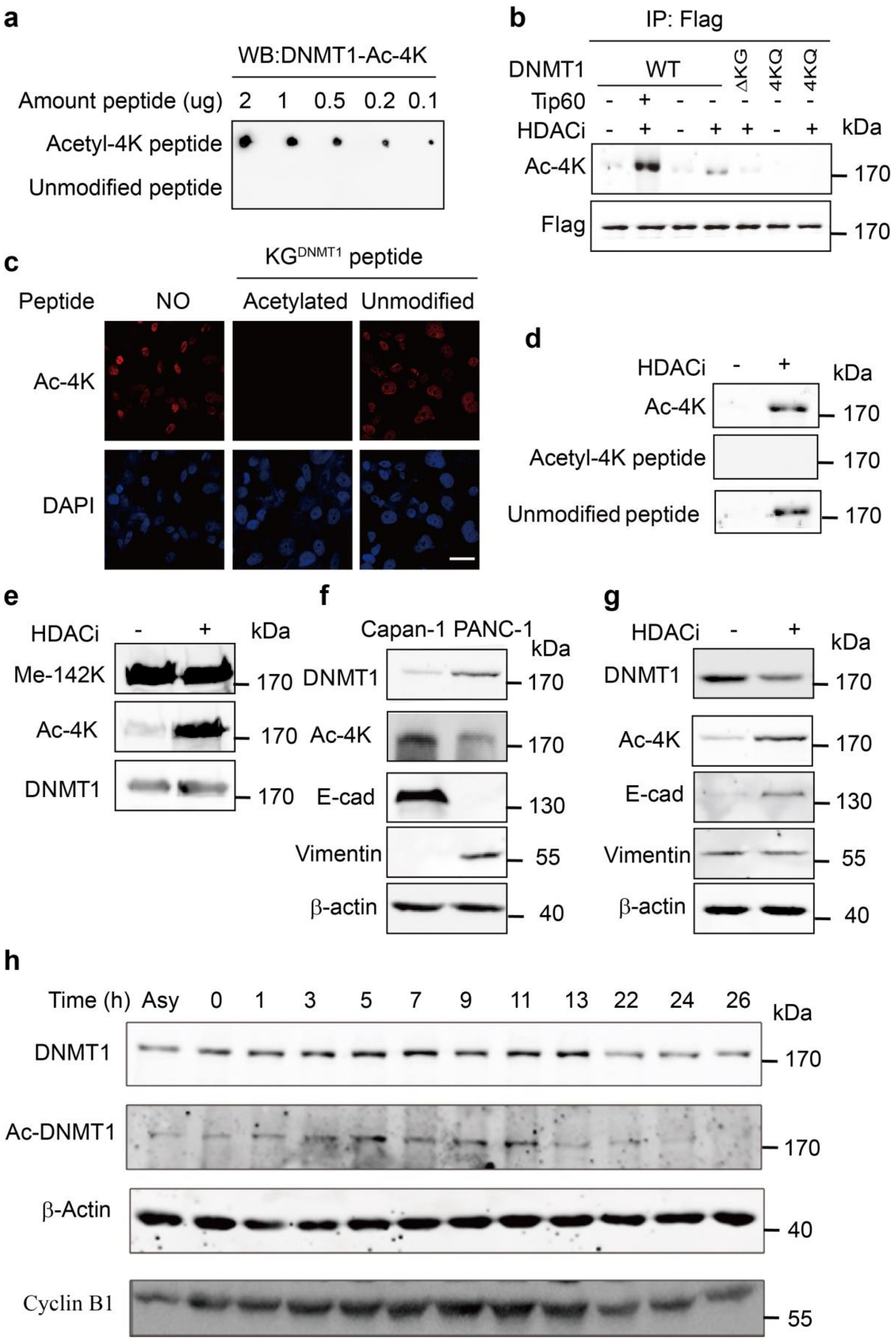

a
WB:DNMT1-Ac-4K
Amount peptide (ug) 2 1 0.5 0.2 0.1
Acetyl-4K peptide
Unmodified peptide
b
IP: Flag
DNMT1 WT ΔKG 4KQ 4KQ
Tip60 - + - - - - -
HDACi - + - + + - +
kDa
Ac-4K 170
Flag 170
c
KGDNMT1 peptide
Peptide NO Acetylated Unmodified
Ac-4K
DAPI
d
HDACi - +
kDa
Ac-4K 170
Acetyl-4K peptide 170
Unmodified peptide 170
e
HDACi - +
kDa
Me-142K 170
Ac-4K 170
DNMT1 170
f
Capan-1 PANC-1
kDa
DNMT1 170
Ac-4K 170
E-cad 130
Vimentin 55
β-actin 40
g
HDACi - +
kDa
DNMT1 170
Ac-4K 170
E-cad 130
Vimentin 55
β-actin 40
h
Time (h) Asy 0 1 3 5 7 9 11 13 22 24 26
kDa
DNMT1 170
Ac-DNMT1 170
β-Actin 40
Cyclin B1 55

Supplementary Figure 6. Characterization of the Ac-4K antibody and negative correlation between DNMT1 and acetyl-DNMT1

(a) Specificity of the Ac-4K antibody determined by dot blot assay. A nitrocellulose membrane was spotted with different amounts of acetylated or unmodified KG^{DNMT1} peptide (corresponding to residues 1109-1119 of human DNMT1). Dot blot assays were performed using the Ac-4K antibody. The Ac-4K antibody specifically recognized the acetylated KG^{DNMT1} peptide but did not cross-react with the unmodified peptide.

(b) Specificity of the Ac-4K antibody against the DNMT1 protein. HEK293T cells were transfected with Tip60 and treated with HDACi. Wild-type or mutant DNMT1 was immunoprecipitated with Anti-Flag M2 agarose, followed by immunoblotting using the indicated antibodies. The result indicates that the Ac-4K antibody specifically recognized acetylated DNMT1, but not $DNMT1^{\Delta KG}$ or $DNMT1^{4KQ}$. Uncropped blots are shown in Supplementary Fig. 7.

(c) Specificity of the Ac-4K antibody for immunofluorescence. Endogenous acetyl-DNMT1 in PANC-1 cells was stained using the Ac-4K antibody preincubated with the acetylated KG^{DNMT1} peptide or the unmodified peptide. Scale bar, 20 μm.

(d) Specificity of the Ac-4K antibody for immunoblotting. HEK293T cells were transfected with Flag-DNMT1 and treated with HDACi. The acetyl-DNMT1 was measured using the Ac-4K antibody or the antibody preincubated with the acetylated KG^{DNMT1} peptide or the unmodified peptide. The acetylated KG^{DNMT1} peptide (but not the corresponding unmodified peptide) completely blocked the signal in immunofluorescence assays and immunoblotting. Uncropped blots are shown in Supplementary Fig. 7.

(e) Methylation of residue K142 of DNMT1 is not affected by addition of HDACi.

HEK293T cells were transfected with Flag-DNMT1 and treated with HDACi. After 48 hours, cells were harvested and analyzed by immunoblotting using the indicated antibodies. Uncropped blots are shown in Supplementary Fig. 7.

(f) Levels of DNMT1 in Capan-1 and PANC-1 pancreatic cancer cells. The protein levels of DNMT1, Ac-DNMT1, E-cadherin, Vimentin, and β-actin were analyzed by immunoblotting using the indicated antibodies. Uncropped blots are shown in Supplementary Fig. 7.

(g) The protein levels of DNMT1, Ac-DNMT1, E-cadherin, Vimentin, and β-actin in Capan-1 cells in the presence or absence of HDACi. Uncropped blots are shown in Supplementary Fig. 7.

(h) The protein levels of DNMT1, Ac-DNMT1, β-actin, and Cyclin B1 in HeLa cells. The cells were collected at indicated time after double thymidine block. Cyclin B1 was used as the marker for M phase of the cell cycle. Notably, no obvious negative correlation between DNMT1 and acetyl-DNMT1 was observed during the cell cycle in HeLa cells. Uncropped blots are shown in Supplementary Fig. 7.

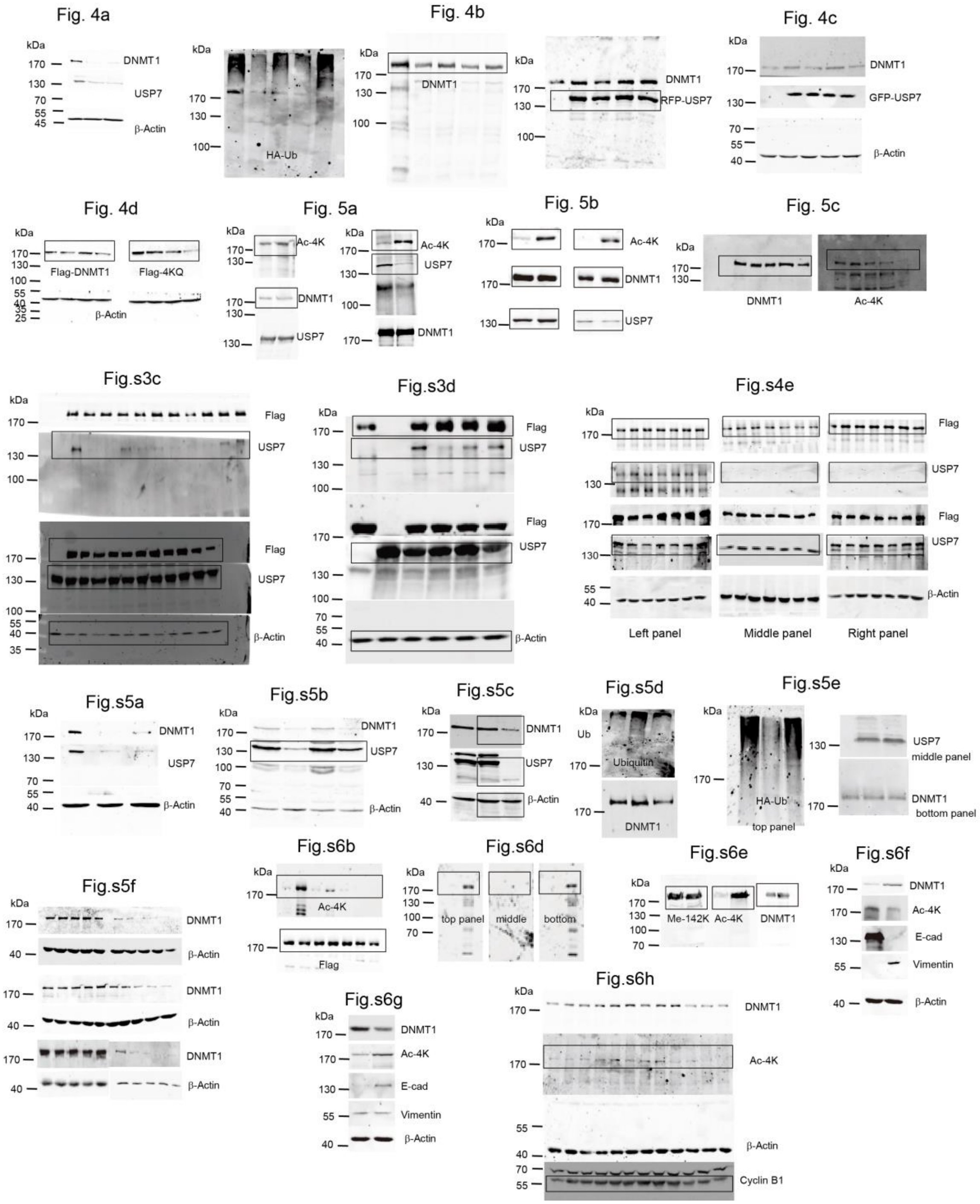

Supplementary Figure 7. Full uncropped figures of western blots. Cropped regions are indicated with rectangles as appropriate.
